# A potent broad-spectrum protective human monoclonal antibody crosslinking two haemagglutinin monomers of influenza A virus

**DOI:** 10.1038/ncomms8708

**Published:** 2015-07-21

**Authors:** Ying Wu, MyungSam Cho, David Shore, Manki Song, JungAh Choi, Tao Jiang, Yong-Qiang Deng, Melissa Bourgeois, Lynn Almli, Hua Yang, Li-Mei Chen, Yi Shi, Jianxu Qi, An Li, Kye Sook Yi, MinSeok Chang, Jin Soo Bae, HyunJoo Lee, JiYoung Shin, James Stevens, SeoungSuh Hong, Cheng-Feng Qin, George F. Gao, Shin Jae Chang, Ruben O. Donis

**Affiliations:** 1 CAS Key Laboratory of Pathogenic Microbiology and Immunology, Institute of Microbiology, Chinese Academy of Sciences, No. 1 West Beichen Road, Beijing 100101, China.; 2 Center for Influenza Research and Early-warning, Chinese Academy of Sciences, No. 1 West Beichen Road, Beijing 100101, China.; 3 Biotechnology Research Institute, Celltrion, Inc., Incheon 406-840, South Korea.; 4 Influenza Division, Centers for Disease Control and Prevention, Atlanta, Georgia 30333, USA.; 5 International Vaccine Institute, Seoul 151-919, South Korea.; 6 Department of Virology, State Key Laboratory of Pathogen and Biosecurity, Beijing Institute of Microbiology and Epidemiology, Beijing 100071, China.; 7 Research Network of Immunity and Health, Beijing Institutes of Life Science, Chinese Academy of Sciences, No. 1 West Beichen Road, Beijing 100101, China.; 8 College of Veterinary Medicine, Guangxi University, Nanning 530004, China.; 9 Office of Director-General, Chinese Center for Disease Control and Prevention (China CDC), Beijing 102206, China.

## Abstract

Effective annual influenza vaccination requires frequent changes in vaccine composition due to both antigenic shift for different subtype hemagglutinins (HAs) and antigenic drift in a particular HA. Here we present a broadly neutralizing human monoclonal antibody with an unusual binding modality. The antibody, designated CT149, was isolated from convalescent patients infected with pandemic H1N1 in 2009. CT149 is found to neutralize all tested group 2 and some group 1 influenza A viruses by inhibiting low pH-induced, HA-mediated membrane fusion. It promotes killing of infected cells by Fc-mediated antibody-dependent cellular cytotoxicity and complement-dependent cytotoxicity. X-ray crystallographic data reveal that CT149 binds primarily to the fusion domain in HA2, and the light chain is also largely involved in binding. The epitope recognized by this antibody comprises amino-acid residues from two adjacent protomers of HA. This binding characteristic of CT149 will provide more information to support the design of more potent influenza vaccines.

## Abstract (web summary)

Monoclonal antibodies with broadly neutralizing activity are being developed as potential treatment of influenza infections. Here, the authors describe a broadly neutralizing antibody with an unusual mode of binding to viral haemagglutinin, which has been isolated from patients convalescent from pandemic H1N1 influenza infection.

## Graphical abstract

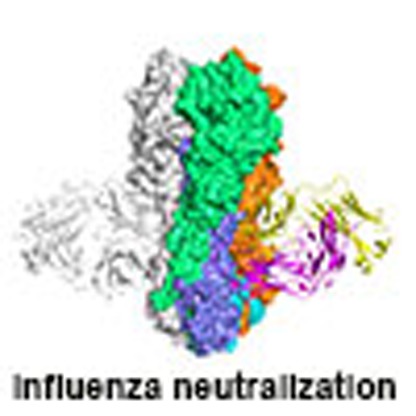

Influenza epidemics of variable severity recur every year with winter season peaks in temperate regions of the world. In addition, four influenza pandemics have been recorded since the beginning of the last century: the 1918 Spanish flu, the 1957 Asian flu, the 1968 Hong Kong flu and the 2009 swine flu^1^,^2^, along with the reemergence of H1N1 virus in 1977 (ref. 3). The impact of these pandemics ranged from an estimated 300,000 to 50 million deaths worldwide for pandemics^4^,^5^. Vaccination is the most effective intervention available to mitigate seasonal and pandemic influenza morbidity and mortality. The current strategy to rapidly immunize the human population against an emerging pandemic relies, for the most part, on quickly adapting to the antigen composition of seasonal influenza vaccines and scaling-up manufacturing as fast as possible. Because massive pandemic vaccination cannot achieve herd immunity until sufficient quantities of vaccine have been produced, pandemic viruses, such as A(H1N1)pdm09, are free to spread for several months. For this reason, pandemic preparedness plans also rely on antiviral medications to mitigate the impact of the pandemic, especially, in the very early stages of the response. Neuraminidase inhibitors are currently the most widely recommended class of antiviral drugs as they are available in stockpiles for immediate use. Unfortunately, some viruses have shown the capacity to develop resistance to these drugs without loss of transmissibility^6^. Therefore, other antiviral small molecules and therapeutic monoclonal antibodies (mAbs) are being developed as alternatives for the treatment of influenza infections^7^.

Passive immunization with plasma-derived antibody products has been suggested and used to treat influenza patients with encouraging results^8^,^9^,^10^,^11^,^12^, although production of hyperimmune sera to influenza viruses is not scalable for wide use in a pandemic response. mAbs produced by immortalized cells in industrial bioreactors would offer an unlimited supply of homogeneous antibody for therapeutic or prophylactic use, yet the highly variable nature of most neutralization epitopes on the influenza hemagglutinin (HA) molecule proposes a problem. This has motivated the search for influenza-neutralizing mAbs that recognize highly conserved neutralizing epitopes on HA. Okuno, *et al*.^13^,^14^ reported a mouse mAb, C179, that showed broad cross-subtype neutralizing activity *in vitro* and protection in mice that were passively immunized. Recent reports indicate that human mAbs targeting a conserved region on the stem of the HA spikes are protective in mouse models of infection^15^,^16^,^17^,^18^,^19^,^20^. These mAbs could be derived from VH and VL (variable region sequences of the heavy-chain (VH) and light-chain (VL)) loci complementary DNA (cDNA) phage display libraries prepared from human immunoglobulin M (IgM)-positive memory cells of influenza-vaccinated or non-vaccinated donors^15^,^16^,^17^. Similar mAbs were also identified by screening phage libraries from non-immune human B cells^18^ or human plasma cells from influenza-vaccinated or infected donors^19^,^20^. Here we present the isolation of a broadly neutralizing antibody, CT149, from convalescent patients infected with A(H1N1)pdm09. We reveal that CT149 exhibits potent neutralization in cell-based tests for divergent HA subtypes and good protection from H1N1, H3N2 and H5N1 subtype viruses and, especially, from the recently emerged human-infecting H7N9 virus^21^,^22^,^23^ in the mouse model. By further structural analysis, we show that the epitope recognized by CT149 is present in the stem region spanning across two adjacent protomers.

## Results

### Isolation and characterization of CT149 mAb

**Figure 1:**
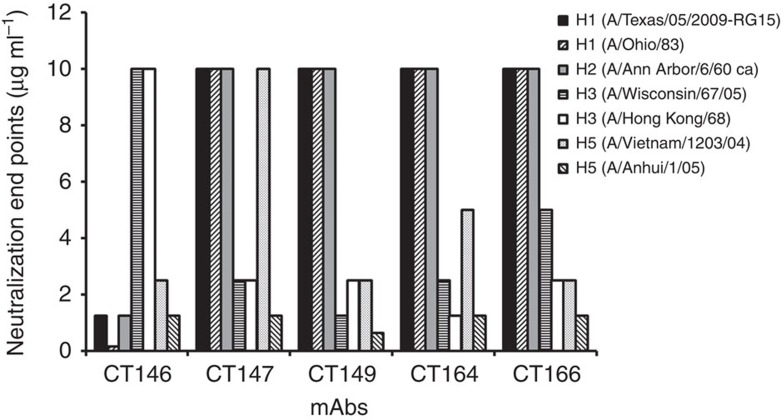
Neutralization activity of human mAbs against different influenza A viruses. Initial MN assay results against various influenza subtypes with selected mAbs. The initial concentration was 10 μg ml^−1^,and therefore the bars truncated at 10 μg ml^−1^ upper limit denote a neutralization end point >10 μg ml^−1^. The experiments have been performed by three times.

In this study, we used the ISAAC (immunospot array assay on a chip) method^24^ using peripheral blood mononuclear cells (PBMCs) from convalescent patients infected with A(H1N1)pdm09 to screen mAbs that might protect against heterosubtypic influenza A virus infection. The mAbs generated directly from single human B cells were screened by enzyme-linked immunosorbent assay (ELISA) against groups 1 and 2 HAs (A/California/04/2009 (H1N1)pdm09 (CA/09) and A/Brisbane/10/2007 (H3N2) (BR/07), respectively). Three of the mAbs (CT149, CT164 and CT166) neutralized representative subtype H5N1 and H3N2 viruses, but failed to neutralize representative H1N1 and H2N2 viruses in an *in vitro* microneutralization (MN) assay (Supplementary Table 1 and Fig. 1). Although the mAbs neutralized various influenza viruses, they failed to inhibit the haemagglutination of turkey erythrocytes by BR/07 virus at concentrations of up to 20 μg ml^−1^ (Supplementary Table 1). These results imply that the antibodies bind in the HA stem region and/or the adjacent region of the globular head, away from the receptor binding site. CT149, which showed the greatest neutralization potency among the three mAbs (Fig. 1), revealed cross-neutralizing activity against virus subtypes with groups 1 and 2 viruses.

Database searches with the VH and VL nucleotide sequences from mAb CT149 (Supplementary Fig. 1) indicated 88.07 and 91.49% of identity to IgHV1-18*01 and IgKV3-20*01, respectively. Several broadly neutralizing anti-HA antibodies have been reported previously such as CR6261, CR8020, F10, FI6 and CR9114 (refs 17, 18, 19, 25). Most of them are originated from the IgHV1-69 germline locus and bind to the stem region of HA only with their heavy chain, mainly using its hydrophobic CDR2. However, FI6 and antibody 3.1, which are derived from IgHV3-30, use both heavy and light chains to bind HA^19^,^26^. Notably, both CT149 and CR8020 derive from the VH1-18 germ line, but CR8020 is specific for group 2 HAs, whereas CT149 can neutralize from groups 1 and 2 HA subtypes. Thus, CT149 probably binds differently to HA, compared with CR8020.

### CT149 neutralizes divergent HA subtypes

To investigate the breadth of the broadly neutralizing antibody CT149 against divergent H3 HAs, 10 H3N2 viruses isolated between 1968 and 2007 were tested in MN assays. CT149 neutralized infectivity by all tested H3N2 isolates, although the neutralization potency varied by nearly two orders of magnitude (0.156–5 μg ml^−1^; Table 1). CT149 also showed neutralizing activity against some tested viruses of group 1 HA subtypes (one H1N1pdm09 virus among the six H1N1 tested, the two H5N1 viruses and two H9N2 strains) and all tested viruses of group 2 (Table 1). Of note, CT149 neutralized two representative subtype H7N9 viruses, A/Anhui/1/2013 (AH1) and A/Shanghai/2/2013 (SH2), which have caused hundreds of severe and fatal human infections since March 2013 in China^27^,^28^,^29^,^30^,^31^,^32^.

The binding affinity of CT149 for different HA subtypes was determined by surface plasmon resonance using soluble HAs. CT149 binding to H1 was between 30.6 and 345 nanomolar (*K*_d_=3.45 × 10^−7^– 3.06 × 10^−8^ M), whereas CT149 bound with higher affinity to H3 HAs, with apparent *K*_d_ values ranging from 1.81 × 10^−9^ to 4.56 × 10^−11^ M. CT149 showed similar binding affinities for H5 and H7 recombinant HAs (rHAs) tested with *K*_d_ values of 2.94 × 10^−9^ and 1.83 × 10^−10^ M, respectively (Table 2 and Supplementary Fig. 2).

Recombinant CHO (Chinese Hamster Ovary) stable cell lines constitutively expressing a full-length HA of CA/09, A/Japan/305/1957 (H2N2), BR/07 and A/Vietnam/1203/2004 (H5N1) (VN/04) were developed to evaluate CT149 in a low pH-induced cell fusion–inhibition (multinucleated syncytium) assay. CT149 inhibited cell–cell fusion of recombinant cells expressing H1, H3 and H5 HAs, but not H2 HA (Supplementary Fig. 3), whereas an isotype-matched negative control mAb (CT-P6) had no effect on cell–cell fusion by any of the four HA subtypes analysed. These observations correlated with the observed neutralization activities of CT149.

### Structures of CT149/H3 and CT149/H7 complexes

**Figure 2:**
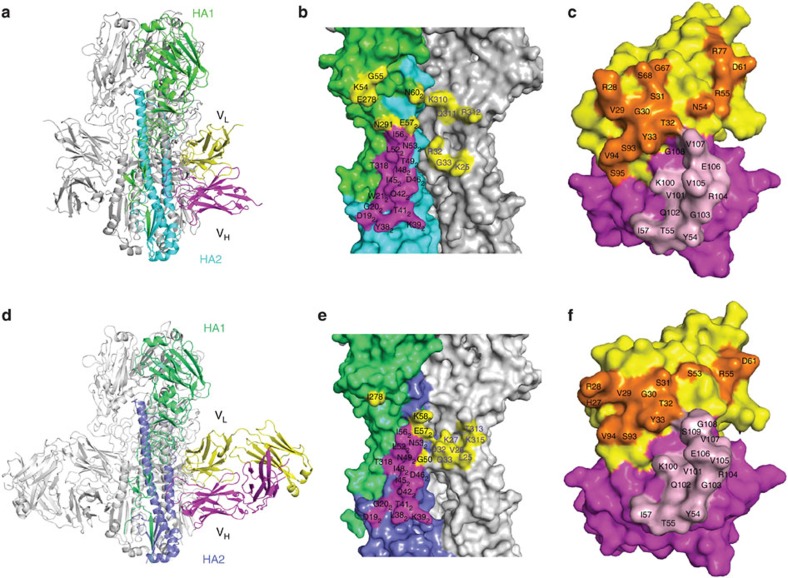
Structures of CT149/H7 and CT149/H3 complexes. The overall structures of CT149/H7 (**a**) and CT149/H3 (**d**) complexes are displayed in a cartoon representation. The antibody CT149 binds the stem regions of both H7 and H3 HA. The epitope residues in H7 (**b**) and H3 (**e**) are denoted in black characters. The purple text refers to the epitope residues in the neighbouring protomer. Residues contacted by the CT149 heavy chain are coloured magenta in both surface representations, whereas residues contacted by the CT149 light chain are coloured yellow The residues of CT149 responsible for the HA binding in the CT149/H7 complex (**c**) and in the CT149/H3 complex (**f**) are marked in black characters. The heavy chain is coloured in magenta and the light chain is coloured in yellow. The residues contacting the HA are coloured in pink for the heavy chain and coloured in orange for the light chain.

The epitope recognized by CT149 was likely to be located in the HA stem region, based on the inhibition of syncytia formation (a surrogate for virion–endosomal membrane fusion; Supplementary Fig. 3), but not haemagglutination (measures receptor function interference by antibody binding to the HA globular head; (Supplementary Table 1). CT149 Fab fragments were prepared and co-crystallized with H3-HA (A/Hong Kong/1/1968) and H7-HA (A/Anhui/1/2013). Final statistics for data collection and structure refinements indicated a resolution of 2.9 and 3.6 Å, respectively (Table 3). Crystal structures of CT149/H3 and CT149/H7 complexes revealed that CT149 indeed recognized residues in the stem region of HA (Fig. 2). In both structures, each HA trimer was decorated by three CT149 Fab molecules (Fig. 2a,b). The heavy chain of CT149 binds into a shallow groove in the fusion subdomain of a single HA protomer, whereas the light chain binds to two regions in separate protomers, one consisting of residues in and near the extended loop of HA2 located in the same HA protomer engaged by the heavy chain and the other consisting of HA1 residues in the fusion domain of the neighbouring HA protomer (Fig. 2c,d and Supplementary Fig. 4). While phylogenetically both H3 and H7 HAs belong to group 2 HAs^33^, and the epitope residues contacted by the CT149 heavy chain were quite similar between these two subtypes, the epitope residues contacted by the light chain of CT149 were moderately different (Fig. 2c,d). The heavy-chain paratopes in the CT149/H3 and CT149/H7 complexes were remarkably similar, whereas the light-chain paratope responsible for HA binding was larger in the H7 complex than in the H3 complex (Fig. 2e,f).

For the interaction between the heavy chain of CT149 and HA, the HCDR3 and HCDR2 loops were found to bind into the shallow groove (as described in ref. 18, and the groove is formed by residues from helix A of HA2, comprising K39, T41, Q42, I45, D46, I48, L52, N53 and I56, as well as Y38 and T49 in H7 or L38 and N49 in H3; (all the residues are in H3 numbering), the HA2 turn (D19, G20 and W21 in H7, or D19 and G20 in H3) and one strand of HA1 (T318) (Fig. 2c,d and Supplementary Fig. 4). The HCDR3 loop was observed to cross-helix A, at an angle of ∼90 degrees, enabling residues V101, V105 and V107 to create hydrophobic interactions with residues in the groove (Fig. 2 and Supplementary Table 2a,b). In addition, the HCDR2 loop was found to contact the N terminus of helix A, enabling residue Y54 to make hydrophobic contacts with residue 38 (L in H3, Y in H7) located at the bottom of helix A (Fig. 2c–f and Supplementary Table 2a,b). Other than hydrophobic interactions, potential hydrogen bonds and polar interactions may also be formed in the contacting site.

For the interaction between the light chain of CT149 and HA, the LCDR1, LCDR2, LCDR3 and FR3 loops were observed to form fewer contacts with the residues in the HA, as compared with the heavy chain (Supplementary Table 2a,b). The residues contacted by the light chain are not conserved between CT149/H3 and CT149/H7 complexes, as a consequence of the divergence between these HA subtypes. In the CT149/H7 complex (Fig. 2c and Supplementary Table 2a), the LCDR1 and LCDR3 loops contact residues from helix A of HA2 on the fusion subdomain (N53 and E57) and also residues from HA1 (K25, R32, G33, K310, Q311 and R312) of the neighbouring protomer, contributing to the majority of interaction between the light chain and HA. The LCDR2 and FR3 loops contact residues from HA1 (K54, G55, E278 and N291) and a residue from helix A of HA2 on the fusion subdomain (N60). In the CT149/H3 complex (Fig. 2d and Supplementary Table 2b), the LCDR1 and LCDR3 loops contact HA2 residues from helix A on the fusion subdomain (G50, N53, E57 and K58) and also HA1 residues (L25, V26, K27, D32, Q33, T313 and K315) of the neighbouring protomer; the latter contributing to the majority of interface between the light chain and HA. The LCDR2 and FR3 loops contact I278 on the HA1 and K58 from helix A on the HA2 fusion subdomain.

**Figure 3:**
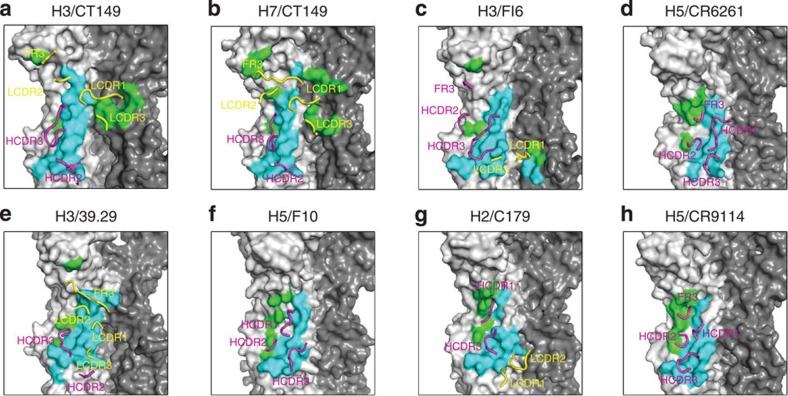
CT149 interactions with H3/H7 and comparison with other stem-bound antibodies. (**a**–**h**)The epitope residues on the HA are displayed in a surface representation. The epitope residues in HA1 are coloured in green and those in HA2 are coloured in cyan. The interacting CDR loops of CT149 are displayed in a cartoon representation. The CDR loops of the light chain are coloured in yellow and those of the heavy chain are coloured in magenta.

Comparison of CT149 with previously reported complexes (for example, FI6, CR6261, 39.29, F10, C179 and CR9114 (refs 17, 18, 19, 25, 34)) revealed that, although the binding sites on HA overlap extensively, important features of the interactions indicate that CT149 binding resembles FI6, 39.29 and C179, which use both heavy and light chains to contact HA, but is markedly different from CR6261, F10 and CR9114, which only use heavy chain to bind HA (Fig. 3). For most previously reported crossreactive antibodies, the light chain of the antibody has contributed only minimally to the binding epitope of these antibodies. Here the light chain of CT149 makes extensive contacts with the HA similarly as 39.29, but in contrast to 39.29, CT149 spans two protomers, whereas 39.29 does not (Fig. 3).

**Figure 4:**
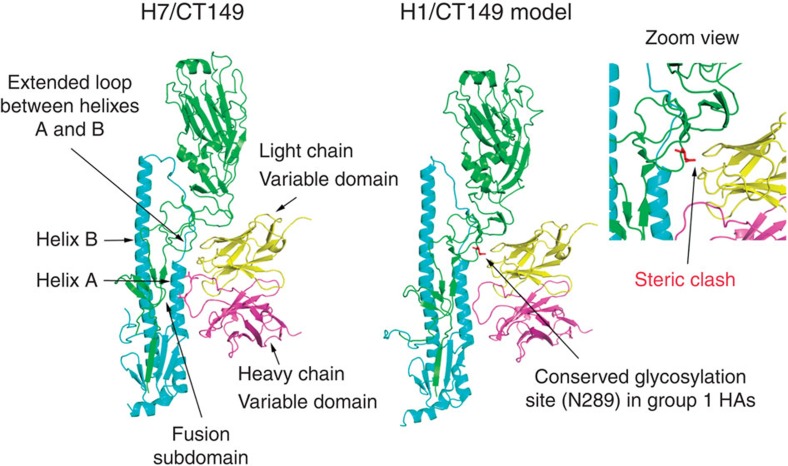
Structural basis for CT149 as a group 2 preferential neutralizing antibody. The light chain of CT149 contacts the region around the extended loop between helixes A and B of HA, and the heavy chain of CT149 contacts the fusion subdomain of HA. By superimposition of H1 and H7 HAs, we have generated a H1/CT149 complex model. There is a relatively conserved glycosylation site in the region around the extended loop of group 1 HA, which could hinder binding of the CT149 light chain.

An important difference between H1 (group 1) and H3 and H7 (group 2) HAs is that the glycosylation status of N289 (HA1); H1 is glycosylated, whereas H3, H7 and all group 2 HAs are not (Fig. 4).A complex glycan linked to N289 in the H1 HA could result in steric hindrance with the CT149 light chain and may well contribute to the observed neutralization differences between these virus subtypes. We should be aware that the steric hindrance by carbohydrate is not a general feature of all group 1 HAs. For example, some H5 and H9 subtype viruses, which are glycosylated at N289, were efficiently neutralized by CT149 as described herein. The light chain of CT149 might interact with H5 and H9 HAs in ways that avoid the steric hindrance from glycosylation at the adjacent N289 (Fig. 4).

### Protection efficacy of CT149 *in vivo*

For *in vivo* protection studies, we chose VN/04 and mouse-adapted A/California/04/2009 (H1N1)pdm09 (maCA/09) as representative viruses from group 1 HA subtypes. AH1 and mouse-adapted A/Hong Kong/1/1968 (H3N2) (maHK/68) were the representatives for group 2 viruses.

**Figure 5:**
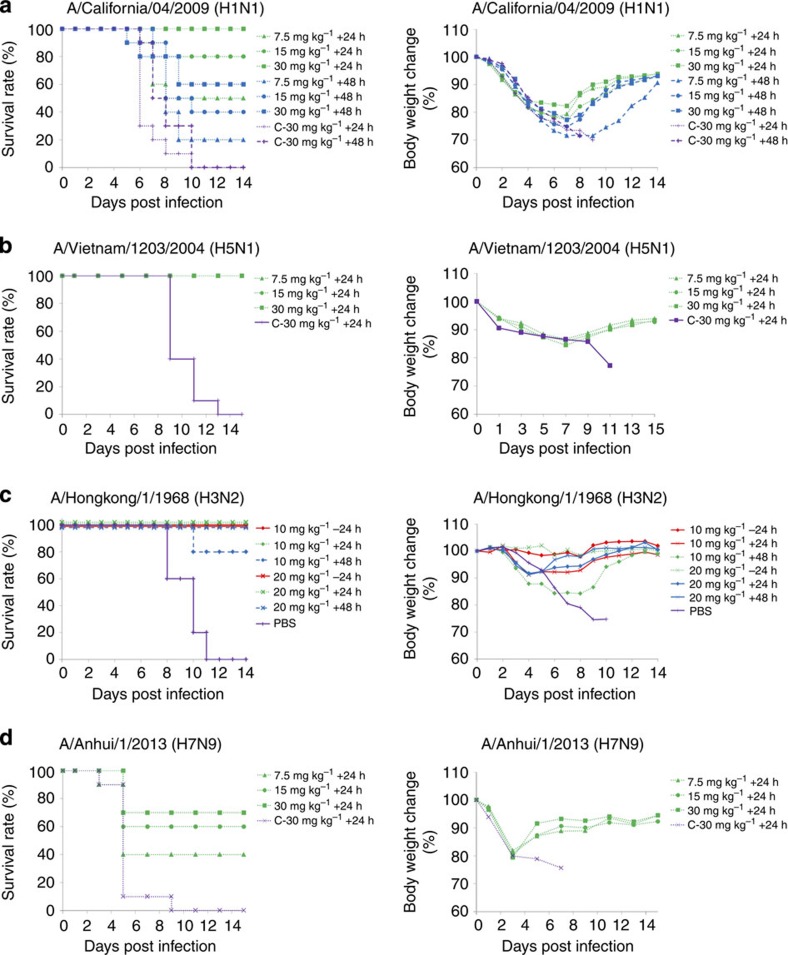
Protection efficacy of CT149 in mice. Mice were infected with a lethal dose of mouse-adapted A/California/04/2009 (H1N1) (*n*=10 per group) (**a**), A/Vietnam /1203/2004 (H5N1) (*n*=10 per group) (**b**), mouse-adapted A/Hongkong/1/1968 (H3N2) (**c**) or A/Anhui/1/2013 (H7N9) (*n*=10 per group) (**d**). The mice were then treated with CT149, isotype-matched negative control antibody (CT-P6) or PBS 24 h before or 24 or 48 h after infection, and surrogate survival end points and body weight changes were monitored for 14 days. Left panels are Kaplan–Meier survival probability curves and right panels represent mean change in body weight per group expressed as the percentage of baseline body weight.

maCA/09-challenged mice (*n*=10 per group) were treated at doses of 30, 15 or 7.5 mg kg^−1^ of CT149 at 24 or 48 h after intranasal virus challenge. Mice treated with CT149 antibody (30 mg kg^−1^ body weight, intraperitoneally) 24 h after maCA/09 virus challenge showed full protection (Fig. 5a). High levels of protection (80% remained healthy) were also evident in mice that were treated with lower doses of the antibody (15 mg kg^−1^ body weight). However, when mice were treated with 7.5 mg kg^−1^ at 24 h after virus challenge, only 50% remained healthy. Furthermore, 60, 40 and 20% of mice treated with 30, 15 and 7.5 mg kg^−1^ of CT149 at 48 h post infection remained healthy, respectively (Fig. 5a). Full protection against highly pathogenic VN/04 was observed when mice (*n*=10 per group) were treated with various doses of CT-P149 (30, 15 or 7.5 mg kg^−1^) at 24 h after inoculation (Fig. 5b).

Mice (*n*=5 per group) treated with CT149 (20 or 10 mg kg^−1^) 24 h before or after virus challenge were fully protected (100% survival) from challenge with maHK/68 (Fig. 5c). Significant levels of protection, 100% and 80%, were also observed in mice (*n*=5 per group) given 20 and 10 mg kg^−1^ of CT149, respectively, at 48 h after inoculation with the maHK/68 virus (Fig. 5c). The *in vivo* protective effect of CT149 against AH1 viral infection was tested in mice (*n*=10 per group) at doses of 30, 15 or 7.5 mg kg^−1^ of CT149 given 24 h after intranasal virus challenge. A single treatment of 30 mg kg^−1^ CT149 in mice provided 70% protection against lethal challenge from H7N9 virus. A lower dose of 7.5 mg kg^−1^ was observed to be partially protective; 40% of mice treated with CT149 survived (Fig. 5d).

Taken together, these results indicated that passive protection by CT149 in mice is dependent on challenged virus, the timing of treatment relative to inoculation and the mAb dose used.

### Fc-dependent activity of CT149

The *in vivo* protective efficacy of CT149 in the mouse model contrasted with its minimal neutralization activity against some of the group 1 influenza viruses tested in the *in vitro* MN assay (Table 1 and Fig. 5a). Immunoglobulin Fc region-mediated effector pathways have been implicated in the protective activity of antibodies *in vivo* by engaging host effector cells in killing virus-infected cells rather than preventing cell infection^35^,^36^. To identify potential host-dependent antiviral functions of CT149, we analysed its activity in antibody-dependent cellular cytotoxicity (ADCC) and complement-dependent cytotoxicity (CDC) assays with CHO cells expressing HA or virus-infected Raji cells. Low concentrations (∼40–200 ng ml^−1^) of CT149 resulted in ∼40% cytotoxicity in the ADCC assay using CHO cells expressing CA/09 HA as targets and PBMCs as effectors, whereas the isotype-matched negative control antibody, CT-P6, achieved only 10% cytotoxicity (Supplementary Fig. 5a). The ADCC effect was also confirmed with virus-infected cells (Supplementary Fig. 5c,d)

The CDC assay with the same target cells revealed that CT149 (∼3 μg ml^−1^) mediated ∼50% cytotoxicity in the presence of human complement (Supplementary Fig. 5b), in contrast to the low cytotoxicity (<10%) levels recorded for isotype-matched mAb, CT-P6.

To evaluate the contribution of Fc-mediated mechanisms to protection in the mouse model of infection, we generated human/mouse chimeric versions of CT149 by exchanging the heavy-chain constant region of CT149 with mouse IgG1 (CT149mIgG1) or mouse IgG2a (CT149mIgG2a). Passive immunization with the chimeric CT149mIgG2a, CT149mIgG1 and the original CT149 revealed protection, albeit at different levels, in animals (*n*=5 per group) that received a low dose (3 mg kg^−1^) of mAb at 24 h post inoculation with an otherwise lethal dose of maCA/09 (Supplementary Fig. 6). The observed trend towards improved protection with CT149mIgG2a is consistent with a postulated Fc-mediated effector function resulting from the higher-affinity Fc-FcγR for IgG2a relative to IgG1 (refs 37, 38). Taken together, these data suggest that CT149 also depends on Fc-mediated effectors for *in vivo* protection as reported for other stem-binding human mAbs^38^.

## Discussion

The identification of broadly neutralizing human mAbs against multiple influenza virus subtypes and the emergence of new subtypes of zoonotic influenza viruses from domestic animals, for example, H7N9, have sparked new interest in the use of antibody therapy in the treatment of severe influenza^39^. The broad and heterosubtypic reactivity of the antibodies is clearly beneficial and suggests that these antibodies could be used as novel antivirals against current and future circulating viruses. In addition, the information obtained from structural analysis of antibody–HA complexes may be used as guides for the rational design of therapeutic molecules and, especially, vaccines^39^.

In this study, we obtained PBMCs from six patients recovered from infection with A(H1N1)pdm09 virus and isolated several mAbs from the single B cells. We focused our attention on CT149 because of its unique profile of reactivity with viruses from both groups 1 and 2. In addition, CT149 was selected particularly owing to its potent efficacy against H3N2 viruses isolated during the past 40 years, and against newly emerging avian influenza A (H7N9) viruses with high propensity for human infections. The neutralization of widely divergent virus subtypes by CT149 indicates that donors infected with A(H1N1)pdm09 can harbour broadly protective mAbs to influenza viruses with groups 1 and 2 HAs as described previously^19^.

The crystal structures of the CT149/HA complexes have confirmed that the mAb recognizes epitopes within highly conserved HA1/HA2 interfaces in the stem. In stark contrast with previously reported broad neutralization epitopes in the stem region, the light chain of CT149 makes extensive contacts with two protomers on the HA^16^,^17^,^18^,^19^,^34^,crosslinking the two monomers. These structural features may help explain the poor *in vitro* neutralization of H1N1 subtype viruses by CT149, even though it was derived from convalescent patients. These viruses feature a highly conserved glycosylation motif for N289 in the HA1 region, adjacent to the extended loop between helix A and B of HA2 (Fig. 4). According to the analysis of H1/CT149 complex model (Fig. 4), we postulate that the N289 glycan may cause steric hindrance by colliding with the light chain of CT149. However, we have not obtained the crystal structure of CT149 in complex with a group 1 HA (for example, H5 or H1), which is a shortcoming to comprehensively elucidate the molecular basis of CT149 neutralization. We cannot rule out the possibility that the N289 glycan may not sterically hinder the binding of CT149 to H1 HA. This should be pursued in the near future.

As the *in vitro* neutralization studies would suggest, mice challenged with maHK/68 (H3N2), VN/04 (H5N1) and AH1 (H7N9) were protected from an otherwise lethal inoculation. In contrast to predictions based on *in vitro* results, CT149 protected mice challenged with maCA/09, VN/04. These observations may be reconciled by considering the Fc-dependent effector functions of CT149 observed in ADCC and CDC assays (Supplementary Fig. 5), as was demonstrated also for FI6 and other mAbs^38^. Recently, CR9114 demonstrated passive protection of mice challenged with influenza type A and B viruses, although this mAb was unable to neutralize type B viruses *in vitro*^17^. These findings in model systems are thought to be medically relevant since human sera from either convalescent patients or vaccinated individuals can induce ADCC of influenza virus-infected cells, presumably mediated by antibodies to HA and NA^35^. Killing of influenza virus-infected cells by CDC with human mAbs was also observed by Terajiman *et al*.^36^. Overall, we found a good correlation between CT149 binding to various HA subtypes, function *in vitro* assays such as neutralization, fusion–inhibition, host factor-mediated killing and *in vivo* protection, but exceptions were noted. For example, the binding affinities to HA epitopes tended to correlate with neutralization potency with some exceptions as previously reported^40^. These differences can be attributed to either intrinsic functional difference between divergent Has, for example, fusion pH optima or host cell specificity, or idiosyncracies imposed by binding and neutralization assay systems. For example, HA density on virions and particle morphology could modulate bivalent interaction of IgG with HA stem epitopes on neighbouring trimers and affect neutralization potency as measured *in vitro*^41^. Similarly, mAb binding assay results could be influenced by undetected structural changes differentially affecting the target HAs^42^,^43^.

Similarly, the overall concordance between CT149 neutralization and low pH-induced polykaryon (syncytia) formation in cells expressing a cognate HA included a notable exception in the case of CA/09 expressing cells. CT149 failed to neutralize the wild-type virus but blocked fusion in this assay, suggesting that the cell-based assay is less stringent than neutralization. Alternatively, differences in HA density, membrane curvature and lipid composition between CHO cell plasma membranes and virions could explain the outcomes of these assays^44^.

To date, a few ‘headless' HA immunogens and chimeric HAs have been designed as potential ‘universal' vaccines that re-direct antibody specificity towards the more conserved regions of the fusion subdomain of the HA stem region^45^. In this study, an antibody that uses both its heavy and light chains to contact the HA could potentially neutralize viruses from divergent groups 1 and 2 HA subtypes, and the breadth of neutralization would be dependent on the interaction between the light chain of antibody and the HA, as the heavy chain usually generates a conserved binding site in the shallow pocket on the fusion subdomain of HA^17^,^18^,^19^,^20^,^25^. This finding may assist in the vaccine design to elicit a more potent crossreactive neutralizing antibody response.

## Methods

### Recombinant HA

The following commercially available monomeric recombinant HAs expressed from both baculovirus and mammalian expression systems were utilized for the studies: A/California/04/2009 (H1N1)pdm09 (Sino Biological, Beijing, China, cat. no. 11055-V08H), A/California/07/2009 (H1N1)pdm09 (Immune Technology, NY, USA, cat. no. IT-003-SW12ΔTMp), A/Texas/05/2009 (H1N1)pdm09 (Immune Technology, cat. no. IT-003-SW16ΔTMp), A/Solomon Island/03/2006(H1N1) (Immune Technology, cat. no. IT-003-0011ΔTMp), A/Ohio/07/2009 (H1N1)pdm09 (Sino Biological, cat. no. 40007-V08H), A/Brisbane/10/2007 (H3N2) (Sino Biological, cat. no. 11056-V08H1), A/Philippines/2/1982 (H3N2) (Immune Technology, cat. no. IT-003-00416ΔTMp), A/Wisconsin/67/X-161/2005 (H3N2) (Immune Technology, cat. no. IT-003-0041ΔTMp), A/Vietnam/1203/2004 (H5N1) (Immune Technology, cat. no. IT-003-0051p) and A/AnhuiAnhui/1/2013 (H7N9) (Sino Biological, cat. no. 40103-V08H). In addition, trimeric recombinant HAs that have foldon trimerization sequences were obtained from the Influenza Reagent Resource (http://www.influenzareagentresource.org): A/California/04/2009 (cat. no. FR-180), A/Brisbane/10/2007 (cat. no. FR-61) and A/Vietnam/1203/2004 (cat. no. FR-39).

### Viruses and cells

Viruses used in this study comprised wild-type isolates and reassortants, containing internal genes from A/Puerto Rico/8/1934 or A/Ann Arbor/1960, which were developed as candidate vaccine viruses for vaccine manufacturing (Table 1). Viruses were propagated in Madin–Darby canine kidney (MDCK) cells or in embryonated eggs. All infectious wild-type H5N1 viruses were handled in Biosafety Level 3 (BSL-3) facilities including enhancements required by the US Department of Agriculture and the Select Agent Program^46^.

For passive protection studies in mice, wild-type A/Vietnam/1203/2004 (H5N1), mouse-adapted A/California/04/2009 (H1N1) and A/Anhui/1/2013 (H7N9) (AN/13) were amplified in embryonated eggs, and mouse-adapted A/Hong Kong/1968 (H3N2) was propagated in MDCK cultures. Virus titres were determined by plaque assay in MDCK cells (plaque-forming unit per ml) or by end point dilution in MDCK cells (TCID_50_) or eggs (EID_50_ per ml).

MDCK (CCL-34) and CHO (CHO-K1 and CCL-61) cells were obtained from the American Type Culture collection (Manassas, VA, USA) and cultured in Dulbecco's modified Eagle's medium (DMEM; Gibco, cat. no. 11965) with or without 10% fetal bovine serum (FBS).

### Isolation of PBMCs from patients recovered from flu

Following laboratory confirmation of influenza A (A/California/04/2009) infection, blood was obtained from patients within 2–4 weeks after the onset of symptoms and was processed immediately. All donors gave written informed consent for research use of blood samples following protocols approved (approval number: 4-2009-0461) by the Institutional Review Board at Severance Hospital, Yonsei University, Seoul, Korea. PBMCs were isolated from the collected blood using lymphoprep (Axis-Shield, Norway, 1114545). The isolated PBMCs were suspended at 2 × 10^7^ cells per ml in KM banker II freezing medium (Cosmobio, Japan, cat. no. KOJ-16092010) and stored in a liquid nitrogen tank for later use.

### Identification and cloning of VH and VL sequences

B cells secreting antigen-specific antibodies were screened using the ISAAC method as previously described^24^. Briefly, the PBMCs were added to each well of the prepared microarray chip at a density of one cell per well. Antibody secretion from single cells was confirmed by binding to pre-coated anti-human IgG antibody. HA-specific antibody-secreting cells were selected with fluorescein isothiocyanate-labelled A/California/04/09 rHA (refs 45, 46, 47). The complete VH and VL of antibodies from each individual antibody-secreting cell were obtained by single-cell 5′-rapid amplification of cDNA ends (5′-RACE), consisting of amplification of an amplicon generated by a reverse transcription–PCR using degenerate primer sets flanking the variable region loci of the heavy and light immunoglobulin chains. Heavy-chain and light-chain DNA amplicons were cloned into pcDNA 3.1(+) expression vectors (Invitrogen, CA, USA, cat. no. V790-20) for fusion with the constant gamma heavy and kappa light-chain coding sequences to prepare expression vectors producing each of the specific immunoglobulins for further characterization. Two chimeric antibodies, CT149mIgG1 and CT149mIgG2a, were also produced via substitution of the Fc region (CH2 and CH3) of the human IgG by those of mouse IgG1 and IgG2a isotypes, respectively, to evaluate the role of Fc-mediated effector functions of CT149 in a mouse model.

### Production of mAbs in mammalian cells

The VH and VL chain genes of selected mAbs were recloned from the pcDNA 3.1-based vectors into the MarEx-based expression vector, pCT107 (patent US 8772021 B2 (2006)), by replacing the heavy and light chains of pCT107 with those from the pcDNA vectors. mAbs for functional evaluation in MN and haemagglutinin inhibition (HI) assays were produced by transient expression. To this end, plasmids encoding mAbs of interest were transfected into human host cell line, F2N78 (ref. 48), and mAbs were harvested by 5 days post transfection and purified by protein A affinity chromatography. Large-scale expression of CT149, for use in animal studies, was performed utilizing stably transfected CHO cells produced by methotrexate selection as described previously (patent US 8772021 B2 (2006)).

### Surface plasmon resonance

Antibody to 6 × His-tag was immobilized to 5,000 resonance units with the amine coupling method on a CM5 chip. His-tagged HA antigen (2 μg ml^−1^ in HBS-EP buffer) was injected for 30 s at a rate of 10 μl min^−1^. Five, threefold dilutions of CT149 (100–1.23 nM) were injected sequentially for 2 min at 30 μl ml^−1^ to record association. After the highest concentration of sample antibody was injected, HBS-EP buffer was injected for 10 min to enable dissociation. *K*_d_ values were calculated from the binding data of all concentrations tested using the Biacore T200 evaluation software set for the bivalent analyte model.

### MN assay

Serial twofold dilutions of mAb (1 mg ml^−1^ stock solution) were prepared, were mixed in equal volumes with 100 TCID_50_ (median tissue culture infectious doses) of the appropriate viruses, were placed in 96-well tissue culture plates and incubated for 1 h at 37 °C. Indicator MDCK cells (1.5 × 10^4^ cells per well) were added to each well and incubated at 37 °C for 20 h. To establish the end point, cell monolayers were then washed with PBS and fixed in acetone, and viral antigen was detected by indirect ELISA with a mAb against influenza A NP (Millipore, cat. no. 18-152). Plates were developed with hydrogen peroxide substrate and tetramethylbenzydine (Sigma) chromogenic reagent. The optical density at 490 nm (OD_490_) was recorded with a plate reader. The virus neutralization titre was defined as the median of reciprocal values of the highest dilutions of antibody yielding ODs below the cutoff value. This cutoff is represented by a 50% specific signal calculated as (virus control OD+cell control OD)/2. The antibody concentration of the end point dilution (titre) represents the median neutralization of the virus analysed.

### Haemagglutination inhibition assay

Virus antigens were mixed with log_2_ mAb dilutions in PBS by dispensing into 96-well plates and incubating at 20–22 °C for 30 min. A 0.5% suspension of turkey erythrocytes was added to each well, and the mixture was incubated for 30 min at 20–25 °C before visual scoring for haemagglutination activity.

### Constitutive expression of HA in CHO cells

The complete coding regions of HA from A/California/04/2009 (H1N1), A/Japan/305/1957 (H2N2), A/Brisbane/10/2007 (H3N2) and A/Vietnam/1203/2004 (H5N1) were synthesized from sequences obtained from the National Center for Biotechnology Information database and cloned into the pMarEx mammalian expression vector (patent US 8772021 B2 (2006)). HA expression plasmids were transfected into CHO-K1 cells using Lipofectamine LTX reagent (Invitrogen, cat. no. 15338-100) following the manufacturer's instructions. Transfected cells were selected with methotrexate^49^,^50^,^51^ and p-clones, without limiting dilution steps (patent US 8772021 B2 (2006)), constitutively expressing HA were identified by immunofluorescence with fluorescein isothiocyanate-conjugated subtype-specific anti-HA antibodies.

### Membrane fusion–inhibition assay

H1, H2, H3 or H5 HA-expressing cells cultured in six-well plates were treated for 5 min with 4 μg ml^−1^ tosyl phenylalanyl chloromethyl ketone (TPCK) trypsin (Sigma, St Louis, MO, cat. no. T1426,) to cleave HA0 into HA1 and HA2, and then quenched with 0.3 ml FBS (final concentration, 10%). The mAbs of interest were added (20 μg ml^−1^) to the wells and cultures were incubated for 1 h at 37 °C followed by medium removal and PBS rinsing. The cells were then exposed to pre-warmed, low pH buffer (150 mM NaCl and 10 mM HEPES, pH 5.0), and incubated for 6 min at 37 °C. The acidic medium was replaced with DMEM supplemented with 10% FBS and cells were incubated for 1 h followed by ice-cold methanol fixation (5 min) and staining with Trypan blue. Syncytium formation was evaluated by qualitatively observing random fields under an inverted microscope (Nikon Eclipse TS100) and photographed using a Digital colour CCD camera.

### ELISA

The binding affinity between mAbs and rHA was measured by ELISA. Wells of 96-well microtitre plates (Nunc, Denmark, cat. no. 449824) were coated with monomeric (A/California/04/2009(H1N1)pdm09 (Sino Biological, cat. no. 11055-V08H), A/Brisbane/10/2007(H3N2) (Sino Biological, cat. no. 11056-V08H1)) or trimeric rHA (A/California/04/2009 (cat. no. FR-180), A/Brisbane/10/2007 (cat. no. FR-61)) (50 μl, 250 ng ml^−1^ in carbonate/bicarbonate coating buffer) and blocked with 1% bovine serum albumin in PBS. Antibodies (threefold dilutions starting from 1 μg ml^−1^) were added and incubated at room temperature for 1 h, and was followed by incubation with horseradish peroxidase-conjugated goat anti-human gamma chain (Zymed, USA, cat. no. 62.8420). After incubation at 1 h at room temperature, the plate was incubated with tetramethylbenzydine (Sigma-Aldrich, MI, USA, cat. no. T0440), and the incubation was stopped by adding 1 N HCl. The absorbance at 450/570 nm was recorded by a plate reader (Spectramax plus 384, Molecular Devices), and the data were plotted with the Prism software package (GraphPad Software Inc., USA).

### ADCC and CDC assays

CHO-K1 cell lines constitutively expressing HA from A/California/04/2009 (H1N1) were used as target cells. Target cells were labelled with calcein-AM (Invitrogen, cat. no. C-3099). The washed cells were plated in triplicate at 1 × 10^4^ cells per well in 96-well plates. The labelled cells were incubated with serially diluted CT149 (from 200 to 0.064 ng ml^−1^) for 30 min. CT-P6, an unrelated mAb with the same IgG1 isotope, was used as the negative control. After incubation of target cells with the antibodies, human PBMCs (the frozen ePBMC, Cellular Technology Ltd OH, USA, cat. no. CTL-UP1) were added to achieve the optimized ratio of effector to target cells of 25:1. Human PBMCs obtained from same donor were used for each experiment. Following 4 h of incubation, arbitrary fluorescent units (AFU) were read on a Spectramax M5 microplate multireader (488 nm excitation/530 nm emission/515 nm cutoff). The maximal ADCC response of CT149 was evaluated as specific % cell lysis by detecting calcein-AM release. Calculation of the percentage of specific lysis from triplicate experiments was performed using the equation of ‘% Cell lysis=(E-S)/(M-S) × 100', where *E* corresponds to the AFU of experimental calcein release, *S* represents the AFU of spontaneous calcein release by target cells in the absence of antibody and *M* equals the AFU of maximal calcein release by target cells on lysis by detergent (2% Triton X-100). For ADCC assay with virus-infected cells, Raji cells^52^ were infected with influenza viruses (A/California/04/09 at 0.03 multiplicity of infection (MOI), A/Perth/16/09 at 0.1 MOI) in OptiPRO SFM (Gibco) for 24 h. The infected cells were stained with calcein-AM (InvitroGen) and washed twice. The stained cells (1 × 10^4^ cells) were added into 96-well plates pre-treated with serially diluted antibodies (CT149 and CT-P6) and incubated for 30 min. The interleukin-2-treated PBMC (the frozen ePBMC, Cellular Technology Ltd, cat. no. CTL-UP1) effector cells were added into the 96-well assay plates at a 25:1 ratio of effector to target cells and incubated for 4 h. The fluorescent signals from samples and controls in triplicate were measured and the cytotoxicity effect of ADCC (%) was analysed as following^53^: cytotoxicity (%)=(mean sample release-mean spontaneous release)/(mean maximum release-mean spontaneous release) × 100.

In the case of CDC, CHO-K1 cell lines constitutively expressing HA from A/California/04/09 (H1N1) were used as target cells. Target cells were plated at a cell density of 3.0 × 10^4^ cells per well in 96-well plates in duplicates and incubated for 16–20 h at 37 °C in a humidified 5% CO_2_ incubator. Following incubation, cells were washed once with 200 μl per well of assay media (20 mM HEPES and 0.1% bovine serum albumin in DMEM/F12(1:1) medium) and filled with 100 μl per well of assay media. An amount of 50 μl of serially diluted CT149 and CT-P6 (from 50,000 to 82 ng ml^−1^) were then added to each well. Thereafter, 50 μl of diluted normal human serum complement (Quidel, San Diego, USA, cat. no. A113) with assay media was added and incubated for 2 h. Finally, 10 μl of cell-counting kit (CCK-8, Dojindo, Kumamoto, Japan, cat. no. CK04) reagent was added to each well and incubated >8 h at 37 °C in a humidified 5% CO_2_ incubator. Absorbance was read on a Spectramax M5 microplate multireader (450–650 nm) to estimate cell viability from the dye-reducing activity levels. The CDC activity of antibodies was reported as cytotoxicity (loss of cell viability) according to the following formula: cytotoxicity (%)=(OD of baseline reaction-OD of experimental reaction)/(OD of baseline reaction-OD of maximal reaction) × 100. In this assay, the baseline reaction value is represented by target cells with complement yet without antibodies. The experimental reaction value is represented by target cells with complement and antibodies, and the maximal reaction value is assessed from target cells lysed in 1% Triton X-100.

### Mice

Virus challenge with mouse-adapted A/Hong Kong/1968 (H3N2) was performed using 6- to 8-week-old female BALB/c mice purchased from Jackson Laboratories. Before the experiment, animals were acclimated for ≥3 days in the Center for Disease Control's (CDC's) animal facility.

For the study using wild-type A/Vietnam/1203/2004 (H5N1), 4- to 5-week-old female BALB/c mice from Samtako (Gyeonggi-do, Korea) were utilized and acclimated for 4 days before conducting the experiment. Housing and care of the animals were performed in the Animal BSL-3 (ABSL-3) facilities located within Bioleaders.

In the challenge experiment using A/Anhui/1/2013 (H7N9), 6-week-old female BALB/c mice (Vital River Laboratories, Beijing, China) were used. Mice were housed in BSL-3 bio-containment cages in the ABSL-3 facilities located in the Beijing Institute of Microbiology and Epidemiology.

Studies using mouse-adapted A/California/04/2009 (H1N1) with CT149 and chimeric CT149 were performed utilizing 9-week-old female BALB/c mice purchased from Orient Bio (Korea). These mice were housed at the pathogen-free facility located at the International Vaccine Institute, Seoul, Korea.

### Protection effects of mAbs on influenza viruses

All animal studies for the evaluation of the protection effects of CT149 on influenza viruses were conducted in compliance with Institutional Biosafety Committee and Animal Care and Use Committee-approved protocols of each facility (approval number for International Vaccine Institute (IVI) is 2012-011, Bioleaders is BLS-ABSL-12-005, Center for Disease Control (CDC) is no. 1854 and Beijing Institute of Microbiology and Epidemiology (BIME) is PBS2013-12). CT-P6 or PBS was injected as the control for all tests at a dose of 30 mg kg^−1^, with exception of the differential passive protection test of the CT149 isotypes, in which 3 mg kg^−1^ of CT-P6 was used as the negative control.

For the differential modulations of the chimeric CT149 isotypes for passive protection with mouse-adapted A/California/04/09 (H1N1), groups of five mice were anaesthetized and inoculated with 5 LD_50_ of virus diluted with 50 μl of PBS. Following abdominal skin disinfection with 70% ethanol, mice were intraperitoneally injected with CT149, CT149mIgG1 or CT149mIgG2a at a dose of 3 mg kg^−1^ body weight. For therapeutic efficacy, groups of 10 mice were injected with one of the three antibody doses (7.5, 15 and 30 mg kg^−1^) at 24 and/or 48 h after inoculation of virus (5 LD_50_) via the intranasal route. Mice were monitored for 14 days and their body weights were recorded. Survival rate was calculated based on the criteria for determining dead mice as either actual death or displaying 30% or greater reduction in body weight loss, which subsequently euthanized.

For the challenge study with A/Vietnam/1203/04 (H5N1), groups of 10 mice were slightly anaesthetized and inoculated with 10 LD_50_ of virus diluted with 50 μl PBS. Beginning at 24 h post infection, 10 mice were treated with CT149 intraperitoneally at doses of 7.5, 15 and 30 mg kg^−1^. Mice were monitored for 15 days and their body weights were recorded. Survival rate was calculated based on the criteria for determining dead mice as either actual death or displaying 25% or greater reduction in body weight loss, which subsequently euthanized.

For challenge with mouse-adapted A/Hong Kong/1968 (H3N2), groups of five mice were slightly anaesthetized by isofluorane inhalation and intranasally inoculated with 50 μl PBS containing 10 LD_50_ of virus. More than 24 h pre-infection or 24–48 h post infection, mice were administered CT149 with a dose of 10 and 20 mg kg^−1^. Mice were monitored for 14 days and their body weights were recorded. Survival rate was calculated based on the criteria for determining dead mice as either actual death or displaying 30% or greater reduction in body weight loss, which subsequently euthanized.

For challenge with an A/Anhui/1/2013 (H7N9), virus was given via the intranasal route (10^6^ plaque-forming unit) before administration of CT149 at a dose of 7.5, 15 or 30 mg kg^−1^ intraperitoneally under anaesthesia. Groups of 10 mice were monitored for survival until 15 days post infection. Survival rate was calculated based on the criteria for determining dead mice as either actual death or displaying 25% or greater reduction in body weight loss, which subsequently euthanized.

### Recombinant HA for crystallization

For production of recombinant H3, residues comprising the HA ectodomain from A/Hong Kong/1/1968 (H3N2) were codon optimized, synthesized, subcloned into the pAcGP67-B baculovirus shuttle vector (BD Pharmingen) and expressed. *Tricoplusia ni* (High 5) cells (Invitrogen) were infected with recombinant baculovirus at an MOI of 5–10 at 28 °C for 72 h. The secreted H3 protein was purified from the tissue culture supernatant by metal affinity chromatography and subsequent size-exclusion gel filtration chromatography (Superdex 200 16/60 column, GE Healthcare). For crystallization, the C-terminal foldon/histidine tag was removed from the H3 proteins by thrombin treatment using 3 U enzyme per mg H3 overnight at 4 °C.

The genes encoding the HA ectodomain from A/Anhui/1/2013 (H7N9) was cloned into the baculovirus transfer vector pFastBac1 (Invitrogen) in-frame with an N-terminal gp67 signal peptide for secretion, a C-terminal thrombin cleavage site, a trimerization foldon sequence and a His6-tag at the extreme C terminus for purification. Transfection and virus amplification were performed according to the Bac-to-Bac baculovirus expression system manual (Invitrogen). H7 proteins were produced by infecting suspension cultures of Hi5TM cells (Invitrogen) for 2 days. Soluble HA was recovered from cell supernatants by metal affinity chromatography using a HisTrap HP 5-ml column (GE Healthcare), then purified by ion-exchange chromatography using a Mono-Q 4.6/100 PE column (GE Healthcare). The purified proteins were subjected to thrombin digestion (BD Biosciences, 3 units per mg HA, overnight at 4 °C) to remove the C-terminal trimerization foldon sequence and His6-tag. For crystallization, the proteins were further purified by gel filtration chromatography using a Superdex 200 16/60 GL column (GE Healthcare) with a running buffer (pH 8.0) of 20 mM Tris-HCl and 50 mM NaCl, and the collected protein fractions were concentrated to 10 mg ml^−1^.

### IgG Fabs for crystallization

CT149 was digested using papain (Roche ref. no.10108014001) protease at an antibody to papain ratio of 100:1 at 37 ^o^C for 1 h. After desalting the column, the material was loaded into a Mabselect Sure column (GE Healthcare, cat. no. 17-5438-03) by applying the flowthrough mode to eliminate the Fc region and undigested antibody. Flowthrough Fab material was concentrated and purified to homogeneity by size-exclusion gel filtration chromatography (Superdex 200 10/300 GL GE Healthcare, cat. no.17-5175-01) with PBS buffer.

### Formation and purification of Fab/HA complexes

CT149 Fab was mixed with purified, His-tag-depleted, recombinant H3 and H7 HA trimers at a molar ratio of five parts Fab to one part HA to ensure saturation with Fab. The resulting CT149 Fab-H3 HA (CT149/H3) and CT149 Fab-H7 HA (CT149/H7) complexes were purified from unbound substrates by size-exclusion gel filtration chromatography (Superdex 200 10/300 column; GE Healthcare) in a buffer comprising 50 mM Tris-HCl (pH 8.0) and 150 mM NaCl or 20 mM Tris-HCl (pH 8.0)and 150 mM NaCl, respectively. CT149/H3 and CT149/H7 complexes were eluted as single peaks between the 158 and 670 kDa molecular weight markers and were concentrated to 12 mg ml^−1^, and used for all subsequent crystallization studies.

### Structure determination of the CT149/HA complex

Initial sparse-matrix crystallization screening was carried out using a Topaz Free Interface Diffusion Crystallizer system (Fluidigm Corporation, San Francisco, CA, USA). Preliminary crystallization conditions for the CT149/H3 complex were obtained after 24 h in several conditions containing the precipitant polyethylene glycol (PEG) 3,000. Following optimization, diffraction quality crystals were obtained at 23 °C using the sitting drop method with 1.0 μl drops containing CT149/H3 in 20% PEG 3,000 and 100 mM Na citrate (pH 5.5). The CT149/H3 complex data set was collected from a single crystal at 3.5 Å resolution at the Advanced Photon Source SER CAT 22-ID beamline. CT149/H3 crystallized in the primitive trigonal space group P3_1_.

The diffraction quality crystals for the CT149/H7 complex were obtained at 18 °C using the sitting drop method with 2.0 μl drops containing 5 mg ml^−1^ CT149/H7 in the no. 11 condition of the Molecular Dimension Screening Kit (MD1-46, Box 1) consisting of 30% (v/v) GOL_P4K, 60 mM Divalents and 100 mM Buffer Systems 3 (pH 8.5). The CT149/H7 complex data set was collected from a single crystal at 2.8 Å resolution at the Shanghai Synchrotron Radiation Facility beamline 17U.

Data collection and refinement statistics are presented in Table 3. Data were processed and scaled using HKL2000 and Denzo^54^. The structures were solved by molecular replacement using Phaser^55^ from the CCP4 programme suite^56^. Initial rigid body refinement was performed using REFMAC5 (ref. 57), and extensive model building was performed using COOT^58^. Further rounds of refinement were carried out using the phenix.refine programme implemented in the PHENIX package^59^ with energy minimization, isotropic ADP refinement and bulk solvent modelling. The structures were then adjusted using COOT and were refined with PHENIX. The stereochemical quality of the final model was assessed with the programme PROCHECK^60^.

The structure of CT149/H3 complex contains an HA trimer and three antibody molecules in the asymmetric unit, and the structure of CT149/H7 complex contain a HA protomer and one antibody molecule in the asymmetric unit. Portion of well-ordered electron density is showed in Supplementary Figure 7.

## Additional information

**Accession codes:**The structures for CT149/H3 and CT149/H7 were deposited in the Protein Data Bank under accession codes 4UBD and 4R8W, respectively.

**How to cite this article:** Wu, Y. *et al*. A potent broad-spectrum protective human monoclonal antibody crosslinking two haemagglutinin monomers of influenza A virus. *Nat. Commun.* 6:7708 doi: 10.1038/ncomms8708 (2015).

## Supplementary Material

Supplementary Information Supplementary Figures 1-7 and Supplementary Tables 1-2

## Figures and Tables

**Table 1:** Neutralization activity of CT149 against viruses from diverse HA subtypes and antigenic drift variants.

Group	Subtype	Virus	MN endpoint (μg ml^−1^)
1	H1N1	A/Ohio/83	>10
		A/Solomon Islands/2006	>10
		A/Ohio/07/2009	5
		A/Texas/05/2009-RG15	>10
		A/Texas/18/2009-RG18	>10
		A/California/04/2009	>10
	H2N2	A/Ann Arbor/6/60 ca	>10
	H5N1	A/Vietnam/1203/04 (VNH5N1-PR8/CDC-RG)	2.5
		A/Anhui/01/2005(H5N1)-PR8-IBCDC-RG6	0.625
	H9N2	A/ck/HK/G9/97(H9N2)/PR8-IBCDC-2	0.312
		A/Green-winged teal/209/TX/2009	0.156
2	H3N2	A/Hong Kong/68	2.5
		A/Philippines/2/1982	0.625
		A/Beijing/353/89-X109-H3N2 PR8 reassortant	0.156
		A/Beijing/32/92-R-H3N2 PR8 reassortant	0.078
		A/Johannesburg/33/94 R-H3N2 PR8 reassortant	0.625
		A/Nanchang/933/95	0.625
		A/Sydney/5/97	0.625
		A/Panama/2007/99	0.312
		A/Wyoming/3/03.rg	5
		A/Brisbane/10/07	0.625
	H7N2	A/turkey/Virginia/2002(H7N2)/PR8-IBCDC-5	10
	H7N9	A/Anhui/1/2013	0.904
		A/Shanghai/2/2013	1.17

HA, hemagglutinin; MN, microneutralization; RG, reverse genetics.

**Table 2:** SPR analysis of CT149 with recombinant HAs.

Subtype	HA donor virus	*K*_d_, M
H1N1	A/California/04/2009*	3.06E-08
	A/Texas/05/2009*	3.38E-08
	A/Solomon Island/03/2006†	3.45E-07
	A/Ohio/07/2009†	5.13E-08
H3N2	A/Philippines/2/1982*	4.56E-11
	A/Brisbane/10/2007‡	1.81E-09
H5N1	A/Vietnam/1203/2004*	2.94E-09
H7N9	A/Anhui/1/2013	1.83E-10

HA, hemagglutinin; SPR, surface plasmon resonance.

^*^Immune Technology

^†^Sino Biological

^‡^IRR

**Table 3:** Data collection and refinement statistics (molecular replacement).

	CT149/H7	CT149/H3
*Data collection*		
Space group	R3_2_	P3_1_
Cell dimensions		
*a*, *b*, *c* (Å)	126.9, 126.9, 409.6	128.7, 128.7, 428.3
*α*, *β*, *γ*(°)	90.00, 90.00, 120.00	90.00, 90.00, 120.00
Resolution (Å)	50.0–2.8 (2.90–2.80)*	50–3.5 (3.63–3.50)
*R*_sym_ or *R*_merge_	11.5 (88.3)	13.6 (71.8)
*I*/σ*I*	17.2 (2.4)	11.9 (1.7)
Completeness (%)	99.5 (99.9)	99.5 (100)
Redundancy	7.8 (7.7)	3.7 (3.9)
		
*Refinement*		
Resolution (Å)	48.4–2.8 (2.90–2.80)	48.3–3.5 (3.50–3.59)
No. of reflections	31,755	94,551
*R*_work_/*R*_free_	26.4/31.1	23.9/27.8
No. of atoms		
Protein	5,650	42,616
Ligand/ion	0	636
Water	25	0
*B*-factors		
Protein	75.313	120.4
Ligand/ion	0	138.4
Water	58.399	0
R.m.s.d.		
Bond lengths (Å)	0.004	0.019
Bond angles (°)	0.780	1.58

R.m.s.d., root mean squared deviation. *Indicates that the values in the parentheses are for the highest-resolution shell.
