# Supplementary material for: A potent broad-spectrum protective human monoclonal antibody crosslinking two haemagglutinin monomers of influenza A virus
Source: Nat Commun. 2015 Jul 21;6:7708. doi: 10.1038/ncomms8708 (PMC4518248; doi:10.1038/ncomms8708)
Supplement: Supplementary Information — Supplementary Figures 1-7 and Supplementary Tables 1-2 [file ncomms8708-s1.pdf]

a

CT149 Heavy Chain

FR1	HCDR1	FR2	HCDR2	FR3	HCDR3	FR4
QVQLVQSGAEVKKPGASVKVSCKTSGYSFS	*TYGVS*	WVRQAPGQGPEWVG	*WISAYTGITDYAQKFQG*	RVTLTTDATTATAFLDLRSLRPDDTATYFCAR	*DKVQGRVEVGSGGRHDY*	WGQGTLVIVSS

b

CT149 Light Chain

FR1	LCDR1	FR2	LCDR2	FR3	LCDR3	FR4
EVVLTQSPGTLALPPGERATLSC	*RASHRVGSTYIA*	WYQQKSGQAPRRLIY	*GASNRAT*	DIPDRFSGSGSGTDFTLTIRRLEPEDSAVYYC	*QQFSVSPWT*	FGQGTRVEIK

Supplementary Figure 1. Amino acid sequence of CT149. Heavy chain (a) and light chain sequence (b) of CT149. Each CDR region is italicized.

A/California/04/2009 (H1N1), Kd = 3.06E-08 (M)

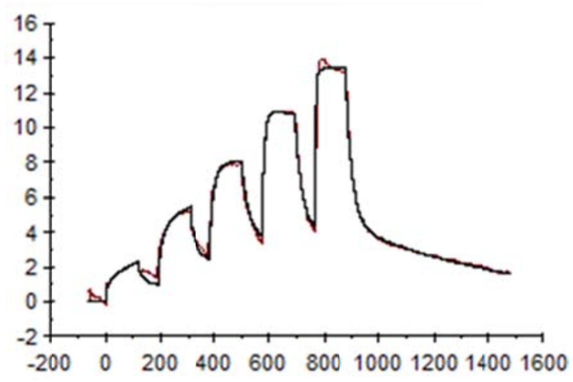

A/Texas/05/2009 (H1N1), Kd = 3.38E-08 (M)

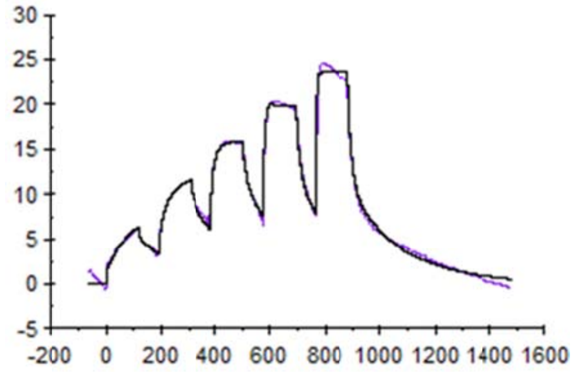

A/Solomon Island/03/2006 (H1N1), Kd = 3.45E-07 (M)

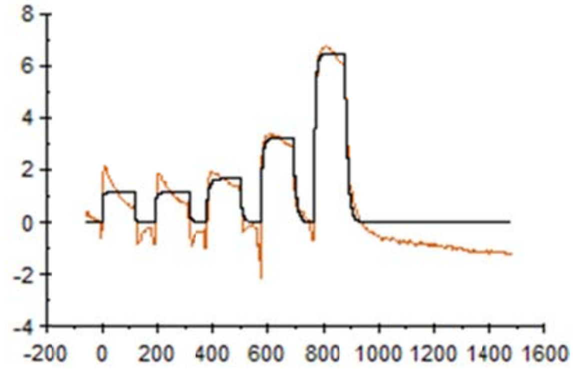

A/Ohio/07/2009 (H1N1), Kd = 5.13E-08 (M)

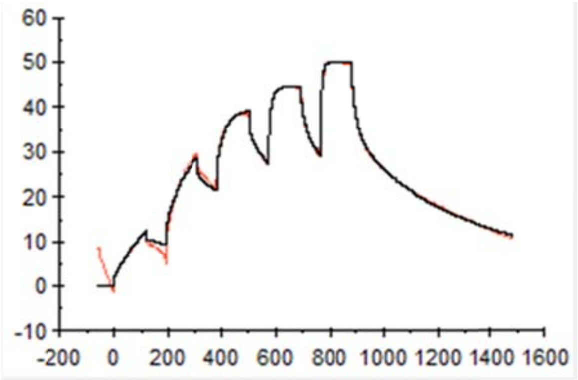

A/Philippines/2/1982 (H3N2), Kd = 4.56E-11 (M)

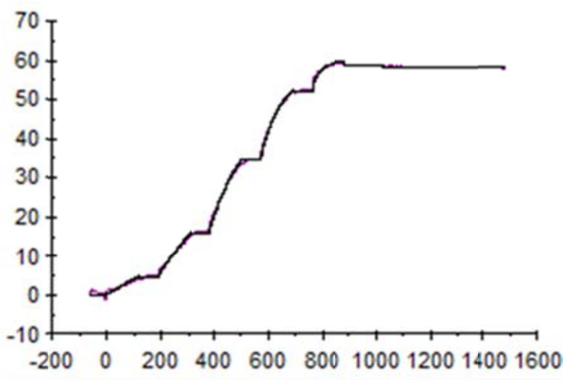

A/Brisbane/10/2007 (H3N2), Kd = 1.81E-09 (M)

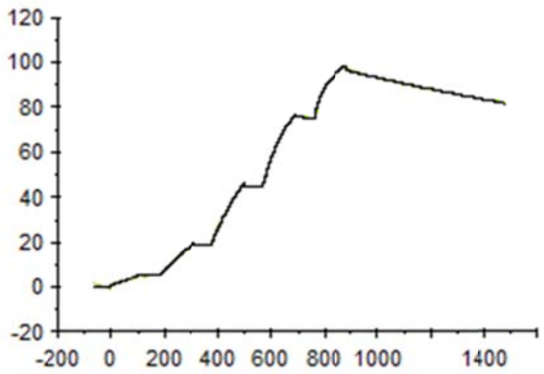

A/Vietnam/1203/2004 (H5N1), Kd = 2.94E-09 (M)

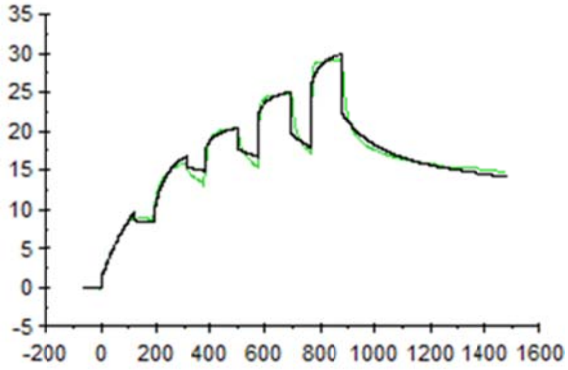

A/Anhui/1/2013 (H7N9), Kd = 1.83E-10 (M)

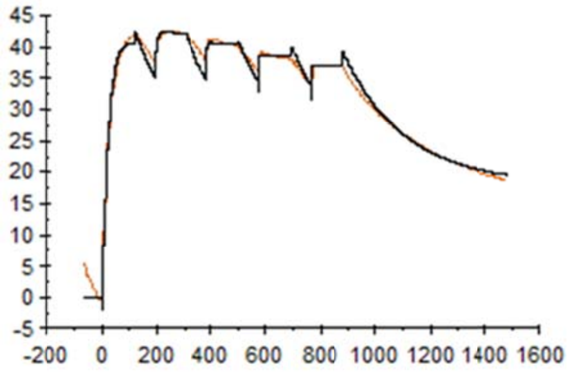

Supplementary Figure 2. Binding curves for reported Kd values of CT-149 for influenza A HAs. Colored curves are the experimental trace obtained from SPR experiments, and black curves are the best fits to the data used to calculate the Kd's represented in Table 2. Single cycle kinetics were used for the SPR method. Kd values were evaluated with the bivalent binding model in Biaevaluation software™.

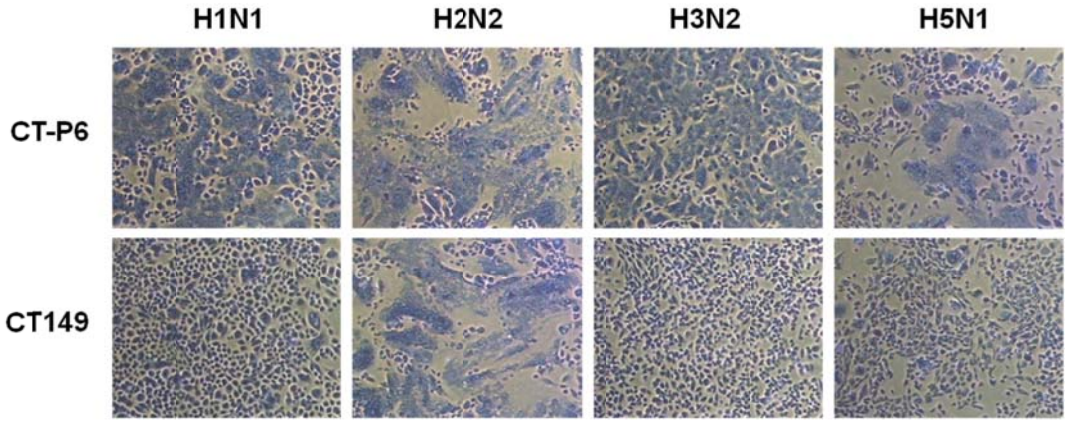

Supplementary Figure 3. Low pH induced cell-cell fusion inhibition assay. CHO cells expressing H1, H2, H3 or H5 HAs from A/California/04/2009 (H1N1) pdm09, A/Japan/305/1957 (H2N2), A/Brisbane/10/2007 (H3N2) and A/Vietnam/1203/2004 (H5N1), respectively, were exposed to low pH buffer in the presence of CT149 or an isotype matched negative control antibody (CT-P6). Representative microscope fields were captured with a digital camera using an objective (10x).

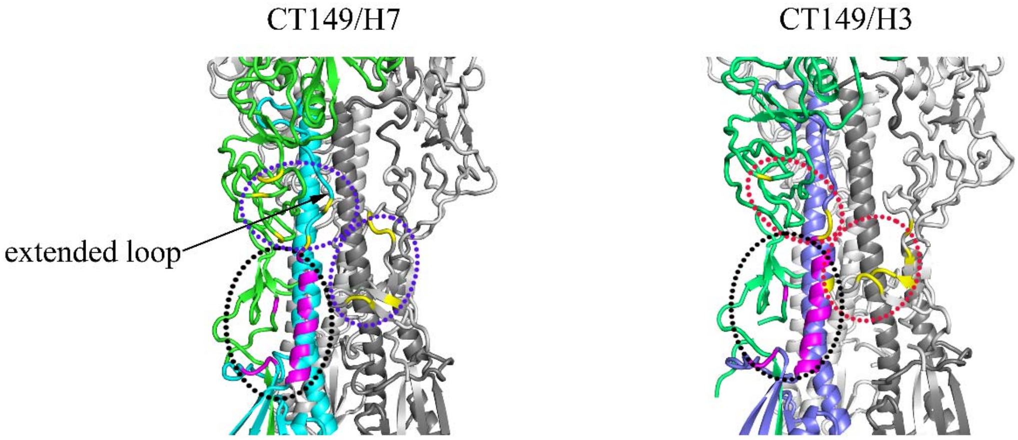

Supplementary Figure 4. Contact region by antibody in CT149/H7 or CT149/H3 complex structures. Contact region by heavy chain is circled by black dashed line, and contact regions by light chain are circled by blue dashed line (in CT149/H7 complex) or red dashed line (in CT149/H3 complex). The residues contacted by heavy chain are marked in magenta, and the residues contacted by light chain are marked in yellow.

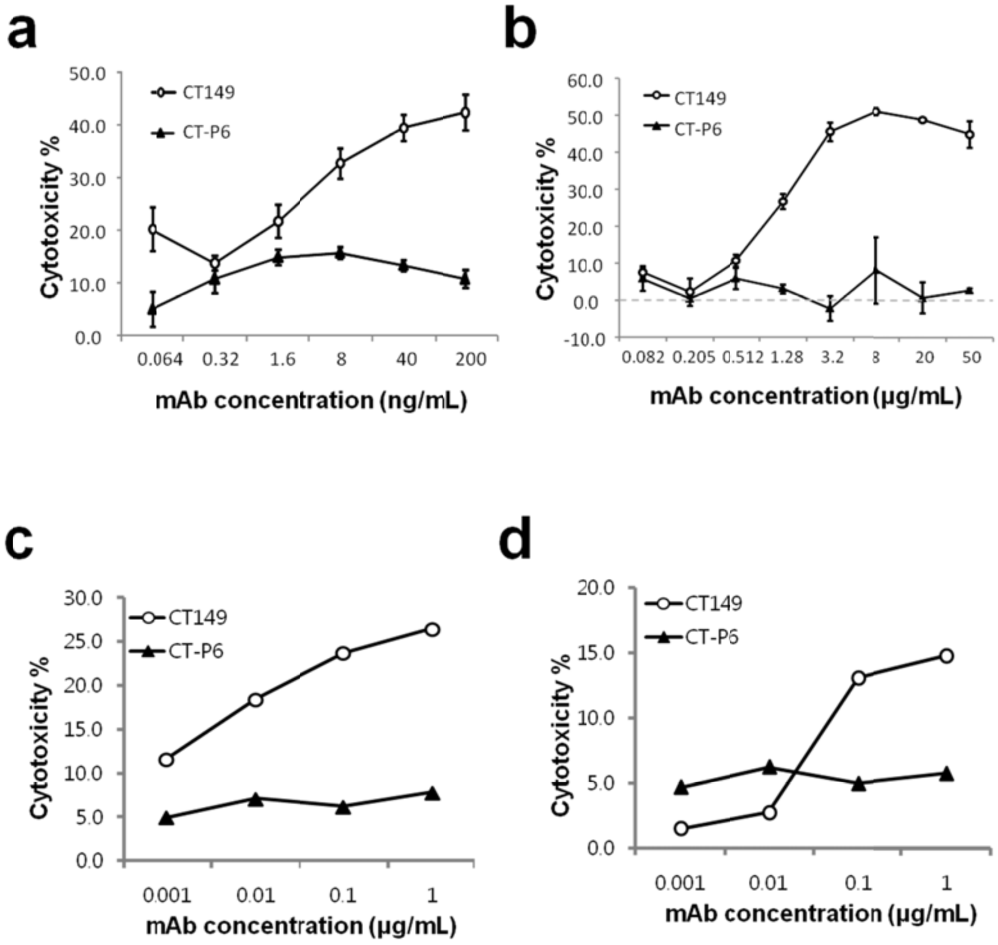

Supplementary Figure 5. Fc-dependent functional assays with CT149. ADCC (a) and CDC (b) effect on CHO cells expressing HA from A/California/04/2009 (H1N1) were measured. ADCC effect on virus infected Raji cells, A/California/04/2009 (c) or A/Perth/16/2009 (d) were measured.

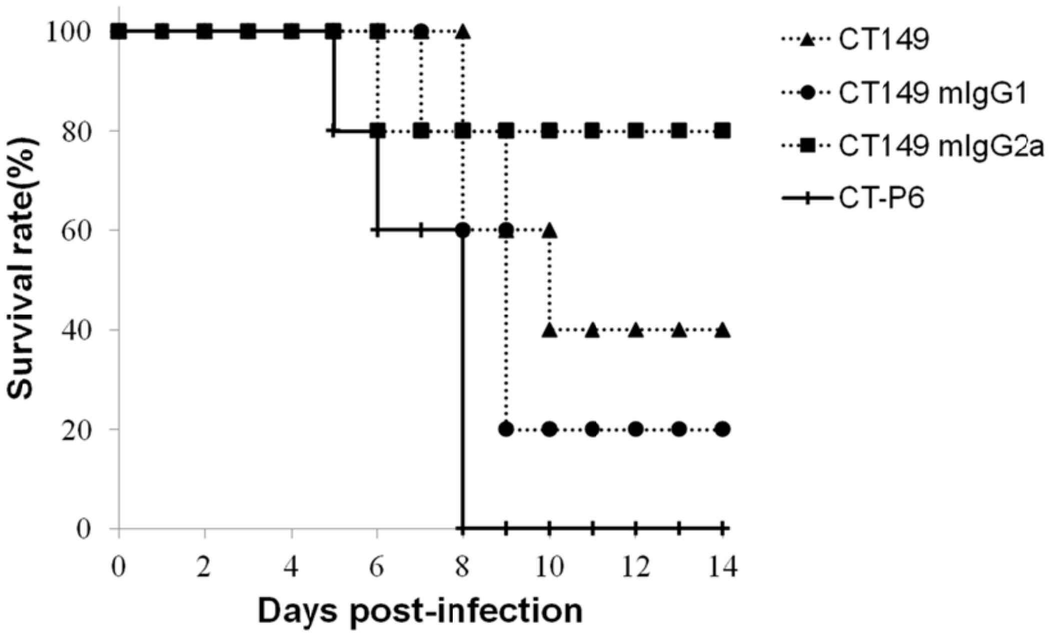

Supplementary Figure 6. Efficacy of human and chimeric CT149 in mice. Therapeutic efficacy of CT149, CT149mIgG1 and CT149mIgG2a with a minimal efficacy dose of 3 mg/kg at 24 hr post viral challenge with mouse adapted A/California/04/2009.

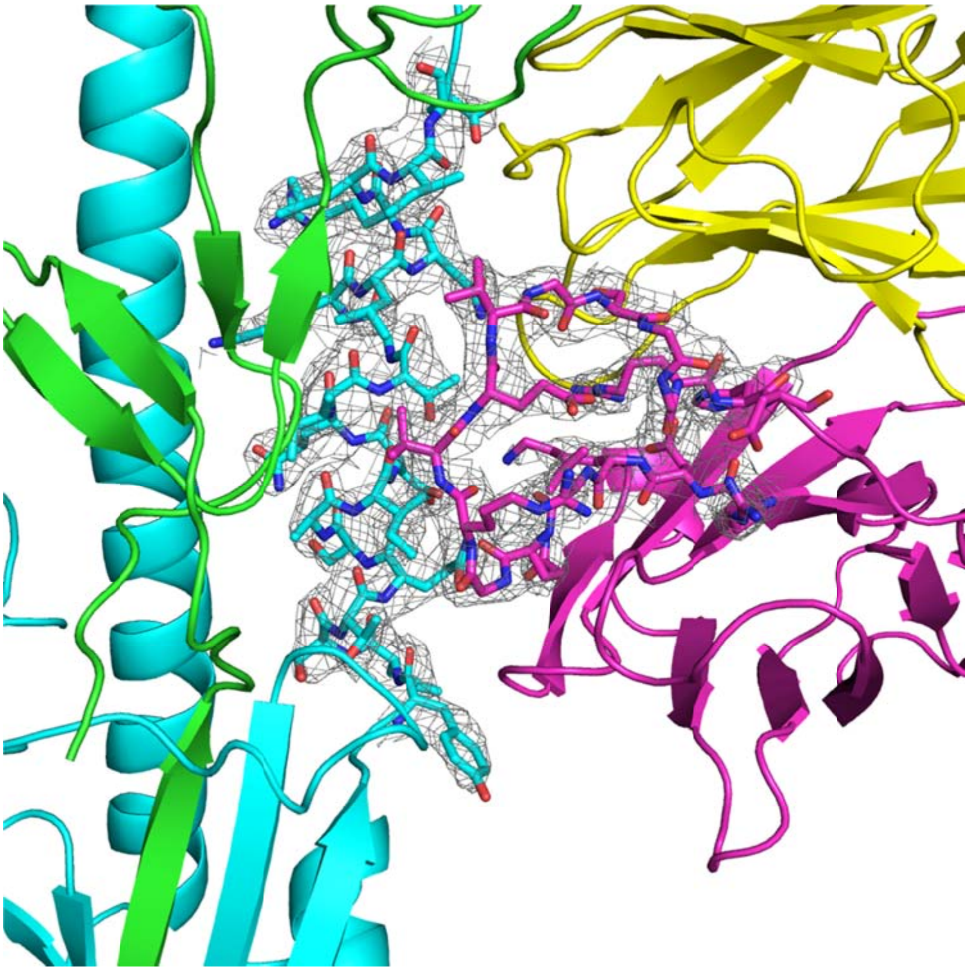

Supplementary Figure 7. Portion of electron density map of CT149/H7 complex structure. The portion of 2Fo-Fc electron density map of CT149/H7 complex structure was contoured at 1.0 sigma, showing the CDR3 loop of heavy chain bound to the helix A of HA2 subunit. The 2Fo-Fc maps were generated by FFT program in CCP4 software, and the figures were drawn by Pymol software.

Supplementary Table 1. Characterization of human mAbs identified by ISAAC

mAb ID[a]	ELISA		MN[b] (μg/ml)				HI	
	H1N1 pdm09	H3N2	H1N1 pdm09	H2N2	H3N2	H5N1 b1/b2	H1[c]	H3 [d]
CT146	+	-	1.25	1.25	>10	2.5/1.25	-	-
CT147	+/-[e]	+	>10	>10	2.5	>10/1.25	-	-
CT149	+/-[e]	+	>10	>10	2.5	2.5/0.625	-	-
CT164	+/-[e]	+	>10	>10	1.25	5/1.25	-	-
CD166	+/-[e]	+	>10	>10	2.5	2.5/1.25	-	-

a. Highest IgG concentration tested was 20 μg /ml
b. MN test with A/Texas/05/2009-PR8-RG15 (H1N1) pdm09, A/Ann Arbor/6/1960 (H2N2), A/Hong Kong/1/68 (H3N2) and b1: A/Vietnam/1203/2004 (H5N1), b2: A/Anhui/1/05 (H5N1).
c. HI test with A/California/07/2009 (H1N1) pdm09 inactivated
d. HI test with A/Brisbane/10/2007 (H3N2) inactivated
e. Very weak positive

Supplementary Table 2a. Interaction between CT149 and H7.

	Antibody	Contacts[a]	HA	
H chain	V105	4, 2	T318,NAG601	HA1 (7)[b]
	V107	1	NAG601	
	Y54	4, 1, 28, 2, 4	D19,G20,Y38,T41,Q42	HA2 (123)
	T55	4, 4, 2	Y38,K39,Q42	
	I57	6	K39	
	K100	3	D46	
	V101	1	Q42	
	Q102	18, 4	Q42, I45	
	G103	4, 3	Q42,I45	
	R104	7	I45	
	V105	2, 3, 1, 3, 1	W21,I45,I48,T49,L52	
	E106	3	T49	
	V107	3, 4, 3	L52, N53, I56	
	G108	5	N53	
L chain	N54	1	N291	HA1 (34)
	R55	9	E278	
	D61	8	K54	
	R77	5, 7, 4	K54,G55,E278	
	S31	4	E57	HA2 (24)
	T32	3, 1	N53, E57	
	Y33	4	N53	
	G67	4	N60	
	S68	8	N60	
L chain	R28	4, 3, 11, 5	R32,K310,Q311,R312	HA1(55) (adjacent monomer)
	V29	3	R32	
	G30	4	R32	
	Y33	6	R32	
	S93	7	R32	
	V94	2, 4, 5	K25, R32, G33	
	S95	1	K25	

a. Numbers represent the number of atom to atom contacts between the antibody residues and the HA residues, which were analyzed by the Contact program in CCP4 suite (the distance cutoff is 4.5 angstroms).
b. Numbers in parentheses represent the total number of atom to atom contacts.

Supplementary Table 2b. Interaction between CT149 and H3.

	Antibody	Contacts[a]	HA	
H chain	R104	1	NAG	HA1 (14)
	V105	8	NAG	
	E106	5	T318	
	Y54	1, 1, 23, 4, 4	D19,G20,L38,T41,Q42	HA2 (160)[b]
	T55	3, 10, 2	L38,K39,Q42	
	I57	3	K39	
	K100	4	D46	
	V101	2	Q42	
	Q102	24, 1	Q42, D46	
	G103	4, 2	Q42,I45	
	R104	2	I45	
	V105	3, 4	I45,N49	
	E106	4, 5, 20, 1	I45, I48, N49, L52	
	V107	11, 4, 12, 1	N49, L52, N53, I56	
	G108	3, 1	N53, I56	
	S109	1	N53	
L chain	R55	2	I278	HA1 (7)
	D61	5	I278	
	G30	3	E57	HA2 (22)
	S31	10, 2	E57, K58	
	T32	2, 1	N53, E57	
	Y33	1, 2	G50, N53	
	S53	1	K58	
L chain	H27	3, 2	L25, Q33	HA1 (52) (adjacent monomer)
	R28	3, 1, 5, 1, 5, 5	L25, V26, K27,Q33, T313, K315	
	V29	2	D32	
	G30	6	D32	
	Y33	8	D32	
	S93	3, 4	D32, Q33	
	V94	1, 3	L25, Q33	

c. Numbers represent the number of atom to atom contacts between the antibody residues and the HA residues, which were analyzed by the Contact program in CCP4 suite (the distance cutoff is 4.5 angstroms).
d. Numbers in parentheses represent the total number of atom to atom contacts.
